# Comparison of patient-reported quality of life outcome questionnaire response rates between patients treated surgically for renal cell carcinoma and prostate carcinoma

**DOI:** 10.1186/s12894-015-0057-y

**Published:** 2015-07-01

**Authors:** David D. Thiel, Andrew J. Davidiuk, Gregory A. Broderick, Michelle Arnold, Nancy Diehl, Andrea Tavlarides, Kaitlynn Custer, Alexander S. Parker

**Affiliations:** Departments of Urology Mayo Clinic, 4500 San Pablo Road, Jacksonville, FL 32224 USA; Health Sciences Research at Mayo Clinic, 4500 San Pablo Road, Jacksonville, FL 32224 USA

**Keywords:** Quality of life, Prostate cancer, Renal cell cancer, Oncology outcomes, Partial nephrectomy, Prostatectomy

## Abstract

**Background:**

We sought to examine differences in response rates to quality of life (QoL) surveys in patients treated surgically for renal cell carcinoma (RCC) and prostate cancer (PCa) and to analyze factors associated with non-response of the surveys.

**Methods:**

Patients who underwent surgery for RCC or PCa between 2006 and 2012 were offered enrollment in respective prospective cancer registries that included baseline and annual QoL assessments. We identified 201 RCC patients and 616 PCa patients who completed a baseline QoL survey and were mailed annual QoL surveys [RCC: SF-36, FACT–G (73 questions), PCa: EPIC, IIEF, Max-PC (80 questions)]. We compared patient characteristics between responders and non-responders using a Wilcoxon rank-sum test for continuous variables and a Fisher’s Exact test for categorical variables.

**Results:**

The overall response rates for the PCa and RCC groups were 63 and 48 % (p < 0.001), respectively. This difference in response rates remained when we limited analysis to only those with early stage disease (pT2 for PCa and pT1 RCC, 62 % vs. 52 %; *p* = 0.03). PCa characteristics associated with response included older age (64.1 vs 62.6 years, *p* = 0.032) and robotic versus open surgery (56 % vs 44 %; *p* = 0.009). There were no characteristics that were associated with response in RCC patients.

**Conclusions:**

Surgically treated PCa patients have higher QoL mail-based survey response rates compared to patients treated surgically for RCC. This difference holds true for clinically localized cancers as well.

## Background

Five-year survival for surgically treated pT1 RCC is over 90 % [1, 2], and 10 year cancer specific survival for surgically treated intermediate risk PCa is over 95 % [3]. A downstream effect of these longer survival times has been a parallel increase in the desire to evaluate factors that affect post-surgical quality of life (QoL). Survey-based instruments to measure specific metrics related to patient QoL (eg, depression, cancer-specific anxiety, etc.) have been developed and validated. These same instruments have been shown to improve physician-patient communication and provide increased individualization of treatment and self-assessment of physician surgical outcomes [4, 5]. Despite the benefit to both research and clinical practice, only about 20 % of urologists report utilizing QoL assessments as part of their management of PCa patients, and patient response rates to these QoL assessments have been shown to vary considerably [6]. This underscores the need to improve our overall understanding of the response rates to QoL assessments in these patient populations and to explore the factors that can predict patient response and non-response.

We harnessed resources at our institution to evaluate response to postoperative QoL surveys in PCa and RCC. We hypothesized that there is a difference in response rates to postoperative QoL surveys between PCa and RCC patients. To test our hypotheses, we utilized data collected as part of two cancer registry efforts at our institution (one for PCa and one for RCC), which include baseline QoL assessment followed by annual evaluations of QoL. We report herein our analysis of the response rates between the two patient populations as well as our assessment of factors associated with response rates for each group, respectively.

## Methods

RCC Patients: Patients who underwent surgery for RCC at our institution between 2006 and 2012 were offered Mayo Clinic Institutional Review Board-approved enrollment in a prospective registry that included baseline and annual QoL assessment. Patients either underwent nephrectomy or partial nephrectomy that was completed laparoscopically or with an open incision. Patients gave written consent and completed QoL surveys at baseline and were mailed follow-up QoL surveys at postoperative year one and two. The QoL surveys mailed were the SF-36 and the FACT-G (Functional Assessment of Cancer Therapy-General). There were a total of 73 questions in the two surveys.

The SF-36 uses 36 questions to assess eight domains of functional health and well-being. It is non-specific to age, disease, and treatment, which is useful in both general and specific populations. All 36 questions on the SF-36 are scored on a scale from 0 to 100, with 100 as the highest level of functioning. Collective scores are calculated as a percentage of the total points possible. The scores from those questions that address each specific domain of functional health status are averaged together for a final score with each of the eight domains assessed [7].

The FACT-G is a 33-item questionnaire that measures four QoL domains; physical, social, emotional, and functional well-being, with nine additional questions dedicated to establishing QoL associated with RCC [8]. The FACT-G is scored by adding the individual scores (range 0 to 108), with higher scores indicating better QoL.

PCa patients: Patients who opted to enroll in the Institutional Review Board-approved PCa registry completed baseline QoL surveys prior to surgical therapy and were mailed follow-up questionnaires 6 months following surgery and then annually thereafter. PCa was treated surgically at our institution with radical retropubic prostatectomy (RRP) or robotic prostatectomy (RARP). The PCa surveys used were the EPIC, IIEF, and Max-PC surveys totalling 80 questions.

The EPIC (expanded prostate cancer index composite) is a prostate-specific instrument utilized to assess health related QoL with regard to function and bother. The instrument assesses urinary, bowel, sexual, and hormonal domains. Three scores are provided for each of the domains to provide a function score, a bother score, and a total score [9]. Higher scores reflect better function.

The IIEF (International Index for Erectile Function) is a brief, reliable, self-administered survey of erectile function that is cross-culturally valid and psychometrically sound. The IIEF addresses the relevant domains of male sexual function (erectile function, orgasmic function, sexual desire, intercourse satisfaction, and overall satisfaction) and has been linguistically validated in 10 languages. The IIEF demonstrates the sensitivity and specificity for detecting treatment-related changes in patients with erectile dysfunction [10].

The Max-PC survey (Memorial Anxiety Scale for Prostate Cancer) was developed to facilitate the identification and assessment of men with prostate cancer-related anxiety. This scale consists of three subscales that measure general prostate cancer anxiety, anxiety related to prostate specific antigen (PSA) levels in particular, and fear of recurrence [11]. It should be noted that patients who did not respond to the questionnaires were not contacted again until the following year. Patients were not contacted by phone or e-mail or sent another questionnaire if there was no response that year.

Study Analysis: We compared patient characteristics between responders (those who returned at least a one- or two-year follow-up survey) and non-responders (those who did not return any follow-up surveys). For the RCC registry, only RCC patients were included. Those with alternative pathology (such as oncocytoma, papillary adenoma, etc.) were excluded. All patients who underwent surgery for PCa were included. The PCa patients do receive a 6 month postoperative survey which was not included in the analysis. The analysis included patients treated surgically between 2006 and 2012 to allow for analysis of 1 year response rates.

Statistical analysis: Continuous variables were presented as median, minimum, and maximum values. Categorical data were presented as counts and percentages. Comparisons of patient characteristics between responders (who completed at least a one- or two-year annual follow-up) and non-responders were performed using a Wilcoxon rank-sum test for continuous variables and a Fisher’s Exact test for categorical variables. The cumulative mortality rates were estimated using the Kaplan-Meier method and comparison between mortality of responders versus non-responders was evaluated using Cox Proportional Hazards models. All statistical tests were two-sided, with threshold of significance set at a = 0.05 and performed using SAS Version 9.2 (SAS Institute Inc., Cary, NC).

## Results

We identified 201 patients in the RCC registry and 616 patients in the PCa registry who were surgically treated between 2006 and 2012 and were asked to fill out a baseline QoL survey and a follow-up QoL survey at 1 year following surgery and annually thereafter. The overall response rates for the PCa and RCC groups were 63 and 48 % (p < 0.001), respectively.

Table 1 outlines the patient and surgical characteristics in the 201 RCC patients stratified by non-response versus response rates. Surgery type (partial nephrectomy or radical nephrectomy), surgical approach (laparoscopic versus open surgery), T stage, or nuclear grade were not significantly associated with increased response rates.

**Table 1:** Association of patient and surgical characteristics in *n* = 201 RCC patients non-response versus response to QoL collected at one- or two-year follow-up

Variable^a^	Non-responder (*n* = 105)	Responder (*n* = 96)	*P*-value^b^
Age at surgery	65.2 (24.0, 87.5)	67.3 (35.3, 92.1)	0.48
Sex, male	75 (71 %)	62 (65 %)	0.36
Surgery type			
Partial	37 (35 %)	44 (46 %)	0.15
Radical	68 (65 %)	52 (54 %)	
Surgery type			0.88
Open	33 (31 %)	29 (30 %)	
LAP	72 (69 %)	67 (70 %)	
T stage			0.14
pT1	69 (68 %)	74 (79 %)	
pT2	9 (9 %)	8 (9 %)	
pT3, pT4	24 (24 %)	12 (13 %)	
Nuclear grade			0.098
1	4 (4 %)	9 (10 %)	
2	62 (60 %)	59 (63 %)	
3-4	37 (36 %)	26 (28 %)	

^a^Median [Minimum, Maximum] is given for continuous measures, and N (%) for categorical measures

^b^P-values for age at surgery and nuclear grade are based on Wilcoxon Rank Sum test. P-values given for Sex, Radical/partial surgical type, Open/LAP surgical type, and T-stage are based on Fisher’s Exact test

RCC = renal cell carcinomaQoL = quality of life

LAP = Laparoscopic

Table 2 summarizes the 616 PCa patients in the PCa registry organized by response status and their association with the type of surgery performed, T stage, and Gleason score. Unlike RCC patients, there was an association with response rates with regard to age at surgery and surgery type (RRP versus RARP). Much like the RCC group, there was no association with response rates and prognostic variables, such as T stage or Gleason score.

**Table 2:** Association of patient and surgical characteristics in *n* = 616 PCa patients versus response to QoL collected at one- or two-year follow-up

Variable^a^	Non-responder (*n* = 230)	Responder (*n* = 386)	*P*-value^b^
Age at surgery	62.6 (29.9, 76.8)	64.1 (42.9, 78.4)	0.032
Surgery type			
RRP	83 (36 %)	171 (44 %)	0.009
RARP	147 (64 %)	215 (56 %)	
T stage			0.51
pT1	0 (0 %)	3 (1 %)	
pT2	200 (87 %)	328 (85 %)	
pT3, pT4	30 (13 %)	54 (14 %)	
Pathological Gleason score			0.71
4-6	86 (37 %)	139 (36 %)	
7	123 (53 %)	211 (55 %)	
8-10	21 (9 %)	36 (9 %)	

^a^Median [Minimum, Maximum] is given for continuous measures, and N (%) for categorical measures

^b^P-values for age at surgery and Gleason score are based on Wilcoxon Rank Sum test. P-values given for T-stage are based on Fisher’s Exact test

PCa = prostate cancer

QoL = quality of life

RRP = Radical Retropubic Prostatectomy

RARP = Robot-Assisted Radical Prostatectomy

Table 3 is a summary of the response rates of stage pT1 RCC patients compared to stage pT2 PCa patients. Despite similar prognoses, the pT2 stage PCa patients are more likely to respond than pT1 RCC patients (62 % vs. 52 % *p* = 0.027).

**Table 3:** Questionnaire return rates of pT1 RCC patients and pT2 PCa patients

Variable	Non-responder (*n* = 269)	Responder (*n* = 402)	*P*-value
RCC pT1 only	69 (48 %)	74 (52 %)	0.027
PCa pT2 only	200 (38 %)	328 (62 %)	

RCC = renal cell carcinoma

PCa = prostate cancer

**Fig. 1:**
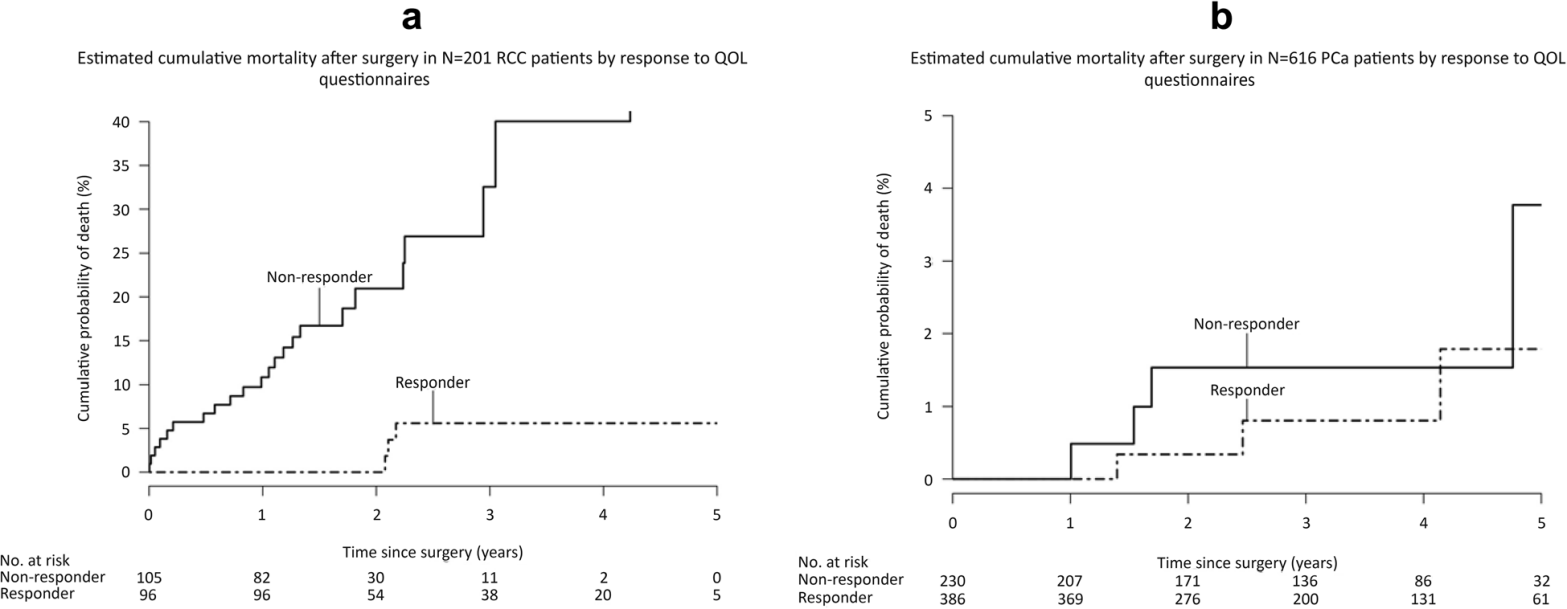
Association of questionnaire return rates with cancer specific mortality: Figure 1**a** demonstrates that 201 RCC patient questionnaire response rates are associated with RCC mortality. Figure 1**b** demonstrates that cumulative PCa mortality following surgery is not associated with response rates

Figure 1a and b demonstrate the survey response rates in relation to cancer specific mortality. It is obvious from both graphs that RCC patients were more likely to die (26 out of 201 RCC deaths) than PCa patients (7 out of 616 PCa deaths). Figure 1a demonstrates that RCC mortality was correlated with non-responder status, and this difference remains even when non-responders who died within the first year after surgery are excluded. Figure 1b illustrates that PCa mortality did not affect questionnaire response rates.

## Discussion

QoL instruments have been shown to improve physician-patient communication, individualization of treatment, and physician self-assessment of surgical outcomes [4, 5]. Despite the advantages of QoL instruments, their use by urologists in addition to overall response rates by patients remains variable [6]. A common complaint regarding QoL instruments utilized for PCa is that these surveys are difficult to seamlessly integrate into practice and often take too much time to score and analyze [6]. Some have argued that these surveys may be too complex for the common patient; however, a recent analysis of the readability of QoL instruments utilized in urology practice for the most burdensome diseases notes that the reading level for these surveys is appropriate for the reading ability of most American adults [4].

A previous analysis of RCC patients at our institution revealed that RCC patients do not necessarily express high levels of concern following surgical treatment secondarily to feeling “cured” [8]. The study noted patients to have mood and anxiety changes early on following surgery, but those changes dissipated with longer-term follow-up. Our QoL instruments are mailed 1 year following surgery, and this time interval certainly allows patients time to recover from surgery and undergo follow-up imaging, possibly re-enforcing the feeling of being “cured.” This may have contributed to the lower mail return rate for the QoL instruments in RCC patients. However, a man who has had a prostatectomy for clinically localized PCa has only a chance of approximately 2-3 % of dying from that disease within a decade, and this would certainly reinforce the feeling of being “cured” [3]. Figure 1 demonstrates that RCC patients are much more likely to die over this short follow-up period than their PCa counterparts. However, when patients with pT1 RCC (who have over 90 % 5 year survival) were compared to pT2 PCa patients in this study, a disparate number of questionnaire responses in favor of the PCa group remained.

One possible reason for the disparity in survey response rates seen in our study between PCa and RCC patients may relate to the treatment decision-making process. RCC patients have few options other than surgery or ablation for their renal mass, which is especially true in clinically localized pT1 tumors. In contrast, patients with clinically localized PCa must decide between a multitude of treatment options such as active surveillance, cryoablation, ultrasound ablation, various radiotherapies, and surgery. The many choices make information gathering paramount and likely underlines the QoL return as a strong focus of PCa follow-up.

A Google search for “Prostate Cancer Treatment Options” currently results in 37 million hits compared to approximately 27 million hits for “Kidney Cancer Treatment Options.” Media exposure and the high prevalence of PCa in aging males may make PCa more socially acceptable and easier to discuss in open forums compared to other cancers. The media exposure and the associated competitive marketing may also be setting men with PCa up for unreal expectations regarding post-therapy outcomes. One analysis demonstrated that men who had a RARP for PCa were more likely to experience treatment regret and dissatisfaction [12]. It is unknown what effect this may have on questionnaire return rates, but patients who had RARP were more likely to respond to QoL surveys than RRP patients.

Another possible reason for the disparity in return rates between RCC patients and PCa patients are the overall operative experiences of the two diseases. There may be a difference in postoperative expectations and QoL perception among patients with RCC and PCa. A diagnosis of RCC or PCa may each carry with it the fear of death. However, patients treated for PCa often carry the additional fear of recovering urinary continence and sexual potency [13]. This fact alone may explain why PCa patients are very in tune with their QoL recovery and with how long it may take to return to baseline QoL parameters. It also must be considered that full recovery of continence and potency following PCa surgery may take up to 24 months [14]. One concern with QoL questionnaire response rates is the length of the surveys. Patients may be unwilling to take the time to fill out lengthy surveys. The PCa and RCC question totals were similar in our registries (80 versus 73 questions) so we do not anticipate that survey length played a role in response rates between RCC and PCa.

One-year QoL response rates in patients surgically treated for PCa are reported as high as 93 % [15]. Two European studies analyzing the QoL of patients following surgery for RCC noted response rates of 71 and 72 % over a 6 month period, with one of the studies utilizing the same SF-36 survey used in this study [15, 16]. As evidenced by the studies above, the QoL response rates in both registries in our study are suboptimal. Our institution is looking into methods that may improve our overall QoL response rates. A 2012 examination of QoL outcomes following renal surgery gave patients the option of internet or paper-based follow-up [17]. Patients who did not respond were contacted by phone or e-mail up to three times. This led to a QoL response rate of 85 % over a 24 week period. Contacting patients via e-mail or phone calls may improve response rates, but it may also lead to inequality of data collection or recall bias, which may influence the results achieved. With regard to PCa, it has been noted that there is a wide gap between patient reported QoL outcomes and those assessed by physicians [18]. Therefore, we believe it is important to continue our current practice of mailing the surveys to the patients. One strategy to explore is direct emphasis to the patient by the surgeon on the importance of QoL survey return. Another option being explored is to deliver QoL survey material during follow-up visits. However, much of our patient population travels a great distance for surgery and receives their cancer follow-up locally, which may make this second option implausible.

Our RCC and PCa registries utilize mail surveys to collect QoL data and one strength of our study is that both surveys are collected in the same manner, which allows us to directly compare QoL return rates. However, this is a single-institution, retrospective study that has a few limitations as a result. One limitation of the study is the small sample size in both registries. This small sample size decreases our power to detect potentially meaningful differences between the PCa and RCC groups. Another limitation is that it involves a sample drawn from a tertiary care center, and the information may not be generalizable to the population as a whole. The respondents in the study were predominantly men, and it is unclear what effect this has on questionnaire response rates. In addition, patient-specific factors such as race and socioeconomic status were not included for analysis and may affect patient response rates. Another factor that may affect response rates that is not recorded is whether or not patients with recurrent cancer are undergoing adjuvant or salvage therapy. Also, it should be noted that the surveys for each population were different, with differing numbers of questions, which may have been another factor possibly impacting survey response rates.

## Conclusions

At our institution, patients who are surgically treated for PCa are more likely to participate in QoL mail surveys than surgically treated RCC patients. QoL response rates for both groups of patients remains suboptimal, and other strategies may be necessary to achieve maximum assessment of postoperative QoL for RCC and PCa patients.

## Abbreviations

EPIC: Expanded prostate cancer index composite
PCa: Prostate cancer
QoL: Quality of life
RARP: Robotic prostatectomy
RCC: Renal cell carcinoma
RRP: Radical retropublic prostatectomy
